# Medical Management of Right Ventricular Dysfunction in Pulmonary Arterial Hypertension

**DOI:** 10.1007/s11897-023-00612-2

**Published:** 2023-07-24

**Authors:** Annalisa Caputo, Silvia Papa, Giovanna Manzi, Domenico Laviola, Tommaso Recchioni, Paolo Severino, Carlo Lavalle, Viviana Maestrini, Massimo Mancone, Roberto Badagliacca, Carmine Dario Vizza

**Affiliations:** Department of Cardiovascular and Respiratory Science, Sapienza University of Rome, viale del Policlinico 155, 00161 Rome, Italy

**Keywords:** Pulmonary arterial hypertension, Afterload reduction, Combination therapies, Right heart reverse remodeling, Pulmonary vascular resistance, Right ventricular overload

## Abstract

**Purpose of Review:**

The purpose of this review is to overview the most relevant and recent knowledge regarding medical management in pulmonary arterial hypertension (PAH)*.*

**Recent Findings:**

Evidence has shown that PAH patients’ quality of life and prognosis depend on the capability of the RV to adapt to increased afterload and to fully recover in response to substantially reduced pulmonary vascular resistance obtained with medical therapy.

Data from recent clinical studies show that more aggressive treatment strategies, especially in higher risk categories, determine larger afterload reductions, consequentially increasing the probability of achieving right heart reverse remodeling, therefore improving the patients’ survival and quality of life.

Remarkable progress has been observed over the past decades in the medical treatment of PAH, related to the development of drugs that target multiple biological pathways, strategies for earlier and more aggressive treatment interventions.

**Summary:**

New hopes for treatment of patients who are unable to achieve low-risk status have been derived from the phase 2 trial PULSAR and the phase 3 trial STELLAR, which show improvement in the hemodynamic status of patients treated with sotatercept on top of background therapy. Promising results are expected from several ongoing clinical trials targeting new pathways involved in the pathophysiology of PAH.

## Introduction

Pulmonary arterial hypertension (PAH) is an obstructive pulmonary vasculopathy that leads to increased pulmonary vascular resistance, right ventricular overload and failure, and to date it is still characterized by high morbidity and mortality rates [1]. It has become clear over the past decade that a more aggressive treatment strategy is fundamental in order to decrease pulmonary vascular resistance (PVR), to prevent right ventricular maladaptation in response to increased afterload, and ideally to achieve right ventricular (RV) reverse remodeling [2].

## Determinants of Right Ventricular Dysfunction in Pulmonary Arterial Hypertension

It is known that PAH patients’ clinical status and prognosis depend mostly on the capability of the RV to adapt to the increased afterload. In PAH, the vascular pathology causes a progressive rise in right ventricular afterload, and the right ventricle progressively increases its contractility in order to maintain its coupling to pulmonary circulation [1].

In fact, homeometric adaptation (i.e., Anrep’s law of the heart) begins within minutes after an increase in the pulmonary arterial pressure (PAP), but when it becomes insufficient in order to support systolic function due to progression of the underlying vascular disease, the right ventricle undergoes heterometric adaptation (i.e., Starling’s law of the heart), with consequential clinical manifestations of RV failure, leading eventually to death [2, 3].

The mechanisms underlying the transition from hypertrophy to dilatation are not fully understood. However, the rate and magnitude of right ventricular overload, as well as the right ventricular adaptation due to genetic factors, neurohormonal activation and right ventricular ischemia supposedly play a role [4, 5].

**Fig. 1:**
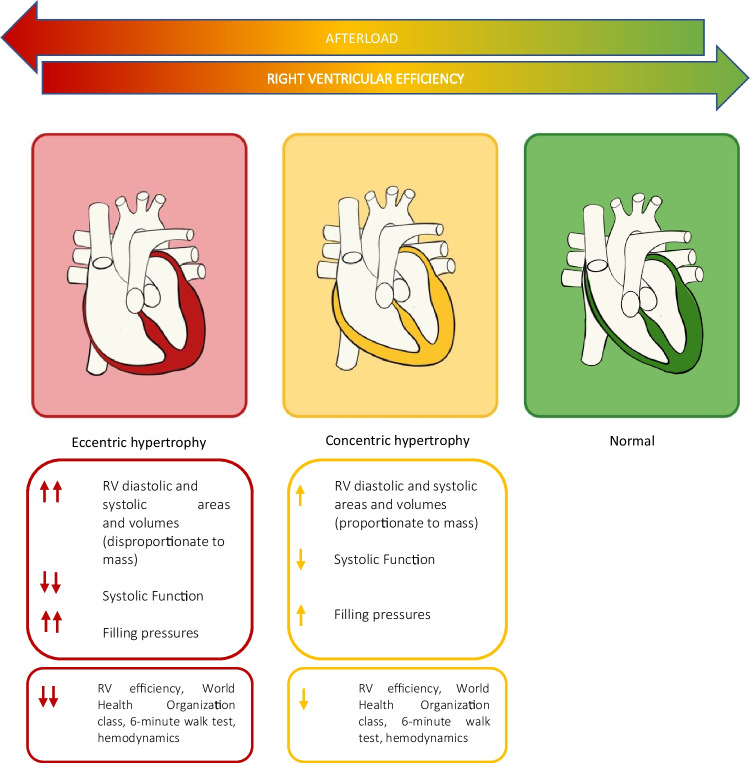
Right ventricular remodeling patterns in pulmonary arterial hypertension

The ability to maintain coupling to pulmonary circulation is also influenced by the adaptive remodeling pattern of the right ventricle (Fig. 1). An eccentric phenotype, expressed by higher RV diastolic and systolic areas and volumes and more elevated filling pressure, seems to be associated with worse WHO class, 6-min walking test, hemodynamic profile and systolic function parameters evaluated with echocardiography and cardiac magnetic resonance (CMR) compared to a concentric phenotype, which seems to be associated with higher RV efficiency confirmed by a higher RV-to-pulmonary arterial coupling [6].

Direct myocardial damage may be also responsible for a more rapid disease progression in specific PAH settings. In HIV-related PAH, inflammatory cytokines and/or viral proteins continuously released by the myocardium might cause an inflammatory damage of pulmonary vessels, characterized by intimal fibrosis and smooth muscle cell proliferation leading to plexiform vascular lesions. In support of this hypothesis is the improved survival of patients with HIV-associated PAH since the introduction of highly active antiretroviral therapy [7].

## The Relevance of Right Ventricular Reverse Remodeling in Pulmonary Arterial Hypertension

Right heart reverse remodeling (RHRR) is considered one of the main goals for clinicians treating pulmonary arterial hypertension. It is defined as a complete morphological and functional recovery of the right heart chambers upon substantial reduction or near normalization of pulmonary vascular resistance obtained with PAH-specific therapy.

An exact clinical definition is still missing, although the assessment of multiple morphological echocardiographic parameters such as RVEDA, right ventricular fractional area change (RVFAC), right atrial area, and left ventricular eccentricity index would be reasonable [2].

Evidence has shown that patients who achieve right ventricular reverse remodeling have an excellent long-term survival and quality of life; however, it can be observed only in a small percentage of patients under treatment [8, 9].

An important clue of the clinical relevance of achieving RHRR can be found in PAH patients undergoing lung transplantation, where early post-transplant normalization of mean pulmonary arterial pressure (mPAP), pulmonary vascular resistance (PVR), and right ventricular ejection fraction was observed during the follow-up period in patients who underwent lung transplantation, regardless of the degree of pretransplant right ventricular dilatation and dysfunction, and tricuspid regurgitation [10].

The impact of RV reverse remodeling on prognosis and survival has been also observed in group 4 pulmonary hypertension patients, whereas near-normalization of PVR achieved after successful pulmonary endarterectomy and balloon pulmonary angioplasty is associated with an important reduction in RV volumes and improvement in RV systolic function [11], with excellent survival compared with patients who did not undergo surgical treatment [12].

Right heart reverse remodeling has recently become crucial for evaluating patients’ response to therapy. In fact, while a mild decrease in PVR achieved with less aggressive treatment strategies is associated with a lower probability of having right heart reverse remodeling, remarkable reductions in PVR associated with more aggressive therapies are associated with more significant improvements in right heart morphological and functional parameters [13].

In a study conducted on treatment-naive idiopathic pulmonary arterial hypertension patients undergoing four different treatment approaches (initial parenteral prostanoid with single oral, initial oral combination, upfront oral combination, oral monotherapy, or parenteral prostanoid monotherapy), the largest reduction in PVR occurred with initial parenteral prostanoid and single oral, with relevant improvement in RVEDA and RV fractional area change observed in patients with a large reduction in PVR (>40%) [13].

This evidence has been confirmed in another cohort of patients with a severe form of PAH study treated with triple upfront combination of targeted therapies, where PVR decreased on average by 69%, and RHRR was observed in all patients, addressing that a time-sensitive and aggressive approach is crucial in order to restore right ventricular function and morphology [14].

## Current Perspectives for Treatment of Pulmonary Arterial Hypertension

Medical treatment for PAH pulmonary is based on three main mechanisms: stimulating the nitric oxide–cyclic guanosine monophosphate (sildenafil, tadalafil, or riociguat), increasing prostacyclin effects on receptors (epoprostenol, Treprostinil, iloprost), agonizing the prostacyclin receptor (selexipag), and antagonizing the endothelin pathway (bosentan and ambrisentan), all leading to vasodilation in the pulmonary vasculature [15].

With the advent of disease-specific treatment, 5-year survival has doubled to more than 60% in the past 30 years. Current treatment consists of combination strategies, targeting more than 1 biological pathway, leading to a noteworthy improvement in morbidity and mortality compared with single-pathway targeted monotherapy [16, 17].

The real turning point that has paved the way for upfront combination therapy in the treatment of pulmonary arterial hypertension has been the AMBITION trial (Ambrisentan and Tadalafil in Patients with Pulmonary Arterial Hypertension). In this trial, the upfront combination of ambrisentan and tadalafil vs either drug alone determined a significant improvement in terms of event-free survival, as well as a 50% decrease from baseline in N-terminal pro-brain natriuretic peptide levels compared with the monotherapy group, a higher percentage of patients with a satisfactory clinical response and a greater improvement in the 6-min walk distance, in patients on double therapy compared to those on monotherapy, while no hemodynamic data was available from the trial [18].

Right heart reverse remodeling is more constantly obtained by combined therapeutic strategies, and the likelihood of right heart reverse remodeling is a sigmoid function of decreased PVR, with > 50% RHRR observed when PVR decreases by > 50% [14].

Upfront combination strategies seem to be more effective in terms of improving hemodynamics and RV morphology and function, and therefore slowing disease progression, compared with oral monotherapy, which is currently recommended by ESC/ERS guidelines only in specific settings of PAH patients [13].

In fact, latest guidelines only recommend treatment with oral monotherapy in patients suffering from cardiopulmonary comorbidities from all risk categories. In this specific setting, patients tend to have a less brilliant response to PAH medication, are more likely to discontinue this medication due to lack of efficacy and tolerability, are less likely to reach a low-risk status, and have a higher mortality risk [19].

Oral monotherapy with riociguat is recommended in patients affected by CTEPH [20].

The positive effects of riociguat have been observed in terms of improved pulmonary artery remodeling, pulmonary hemodynamics, improved RV-PA coupling, improved RV function, reduced RA and RV dimensions, RV hypertrophy and fibrosis, reduced tricuspid regurgitation both in preclinical models and prospective randomized controlled phase III trials such as PATENT-1 and CHEST-1 [20].

Apart from these settings, evidence shows that the effect monotherapy on afterload reduction is mild, with only a slight reduction in PVR after 3 to 6 months of treatment [21].

More encouraging results were obtained in patients treated with high dose treprostinil s.c. and epoprostenol i.v. compared with oral drugs [13, 22, 23].

The use of upfront combination with ERA+PDE5i for the treatment of low and intermediate risk patients without cardiopulmonary comorbidities is recommended by the latest guidelines. It is recommended to maintain this therapy also in patients that during follow-up belong to the low-risk category according to the four-risk strata model [19].

Apart from the AMBITION study, the use of combination therapy is supported by other smaller real-life studies, which have proved that initial oral combination therapy can also significantly improve the hemodynamic status, with PVR reduction of 27–59% and mPAP reduction of 10–23% (from baseline) [14, 24].

A recent multicenter retrospective analysis revealed that in newly diagnosed PH patients undergoing double upfront therapy with ambrisentan and tadalafil, in spite of the observed improvement REVEAL risk score in proportion to decreased PVR and preserved stroke volume, still 50% of these patients did not achieve low risk status and needed additional treatment [25].

In patients who are at intermediate–low risk despite receiving double oral therapy with ERA+PDE5i, adding selexipag should be considered to reduce the risk of clinical worsening, but also switching from PDE5i to riociguat may also be considered [19].

Evidence supporting these treatment strategies comes from randomized controlled trials such as the GRIPHON, where adding Selexipag in treatment naïve patients or on top of background therapy was associated with a reduced risk of clinical worsening events [26] and REPLACE, where enrolled patients were randomized to continue their PDE5i or to switch from a PDE5i to riociguat. In this study, the primary composite endpoint of 6MWD, WHO-FC, and NT-proBNP was met, and clinical improvement at follow-up and fewer worsening events were demonstrated in half of the patients who switched to riociguat [27, 28].

According to the latest guidelines, in patients belonging to the high-risk group at baseline, in patients at intermediate risk presenting with severe haemodynamic impairment (e.g. RAP ≥20 mmHg, CI <2.0 L/min/m^2^, SVI <31 mL/m^2^, and/or PVR ≥12 WU), and in patients presenting at intermediate-high or high risk at follow-up risk assessment, triple combination therapies with ERA+PDE5i and IV/SC PCA are recommended [19].

Several recent observations highlight the therapeutic potential of this strategy. These studies suggest that an upfront triple combination therapy that includes a parenteral prostanoid reduces PVR by 70% and mPAP by 30%, whereas treatment with subcutaneous prostanoid, an endothelin receptor antagonist and phosphodiesterase-5 inhibitor was also associated with right heart reverse remodeling in one of these studies [14, 29].

Evidence derived from case series has shown that this strategy among all has the most relevant impact on mortality [14], and registry data from France showing that initial triple-combination therapy including an i.v./s.c. prostacyclin analogue was associated with better long-term survival compared with monotherapy or dual-combination therapy confirm these results [16]. Given the absence of robust evidence, this strategy is not supported for treatment of lower risk categories, whereas risks may actually exceed substantial benefits [19].

Conversely, initial oral triple-combination therapy is not recommended by current guidelines [19]. Evidence comes from the Efficacy and Safety of Initial Triple Versus Initial Dual Oral Combination Therapy in Patients With Newly Diagnosed Pulmonary Arterial Hypertension (TRITON) study, where both treatment strategies remarkably reduced PVR by week 26, with no significant difference between groups [30].

Next to PVR reduction achieved by PAH-specific treatment, evidence shows the possible role of PD5-I in improving right ventricular systolic function and adaptation to increased afterload; however, more evidence is needed to confirm a direct effect of treatment on the myocardium [31].

## Aggressive and Early Approaches: Improving Hemodynamics and Survival

Observational studies suggest that aggressive treatment strategies, especially including early parenteral prostanoids, seem to improve cardiopulmonary hemodynamics in PAH more efficiently than early yet milder or delayed add-on therapeutic approaches, especially for higher-risk patients [14].

Recent evidence supporting a more aggressive therapeutic approach comes from an Italian study by D’Alto et al., where treatment with triple upfront combination therapy with ambrisentan, tadalafil, and subcutaneous treprostinil in patients with a severe form of nonreversible idiopathic pulmonary arterial hypertension was associated with considerable clinical and hemodynamic improvement and right-sided heart reverse remodeling [14].

In another study, remarkable improvements in terms of hemodynamic status (mPAP and PVR) and excellent long-term survival rates were observed in patients’ with severe PAH, defined by a PAPm >50 mmHg, who received early treatment and rapid uptitration with parenteral prostanoids in combination with background oral therapy [32].

Early introduction and rapid uptitration of parenteral drugs were also proposed by Benza et al., who recently proposed the use of an implantable CardioMEMS to remotely monitor pulmonary hemodynamics and guide titration of parenteral prostacyclins. Within the first months of monitoring, patients underwent rapid uptitration in parenteral prostacyclin, and significant reductions in mPAP and an improvement in cardiac output were observed in response to therapy. Significant function and quality of life improvements were observed in the cohort within 1 year [33].

On the other hand, data from an Italian study by Badagliacca and colleagues showed how late introduction of parenteral drugs was associated with scarce prognosis. This was relatable to the advanced clinical and hemodynamic condition of the patients, proving once again the importance of adopting early and aggressive treatment strategies in order improve the patients’ outcome and life expectancy [34].

## New Hopes for Treatment of Pulmonary Arterial Hypertension

Currently approved PAH therapies, which act via the prostacyclin, endothelin or nitric oxide pathways, have radically modified the course of the disease; however, long-term prognosis remains poor. New therapeutic targets and novel therapies are needed to achieve low-risk status in a higher percentage of patients, and ultimately improve disease prognosis.

Encouraging results have derived from the double-blind, randomized, placebo-controlled trials PULSAR and STELLAR, which evaluated the effect of sotatercept on top of background pulmonary arterial hypertension therapy.

Sotatercept is a novel fusion protein composed of the extracellular domain of the human activin receptor type IIA fused to the Fc domain of human IgG1, acting as a ligand trap for members of the TGF-β superfamily and by balancing the growth-promoting activin growth differentiation factor pathway and the growth-inhibiting BMP pathway.

In the phase 2 PULSAR trial, treatment with sotatercept determined an important reduction in pulmonary vascular resistance compared to placebo. The least-squares mean difference between the sotatercept 0.3-mg group and the placebo group in the change from baseline to week 24 in pulmonary vascular resistance was −145.8 dyn · sec · cm^−5^ (95% confidence interval [CI]; *P* = 0.003), while the least-squares mean difference between the sotatercept 0.7-mg group and the placebo group was −239.5 dyn · sec · cm^−5^ (95% CI; *P* < 0.001). Remarkable improvements in the 6MWT and NT-proBNP levels were also documented. Improvements in primary and secondary endpoints and clinical efficacy were confirmed in the extension period [35].

The outstanding results were confirmed in the phase 3 trial (STELLAR), in which the patients were randomly assigned in a 1:1 ratio to receive subcutaneous sotatercept (starting dose, 0.3 mg per kilogram of body weight; target dose, 0.7 mg per kilogram) or placebo every 3 weeks.

The median change from baseline at week 24 in the 6-min walk distance was 34.4 m (95% confidence interval [CI], 33.0 to 35.5) in the sotatercept group and 1.0 m (95% CI, −0.3 to 3.5) in the placebo group. The first eight secondary end points were significantly improved with sotatercept as compared with placebo [36].

A recently completed phase 2 randomized controlled trial, currently in the open label extension phase, evaluated the effect on improving hemodynamics determined by seralutinib (formerly known as GB002), a novel, potent, clinical stage inhibitor of PDGFRα/β, CSF1R, and c-KIT delivered via inhalation. In preclinical animal models, the drug reversed pulmonary vascular remodeling, improved hemodynamics, reduced circulating levels of N-terminal pro b-type natriuretic peptide (NT-proBNP), and increased lung BMPR2 protein expression compared to controls, while phase 1a and phase 1b trials proved that it was well tolerated [37].

The ongoing phase 2b/phase 3 randomized controlled trial IMPAHCT aims to evaluate safety and efficacy of dry powder inhaled imatinib (AV-101) in patients with group 1 PAH. Imatinib is a small-molecule kinase inhibitor which inhibits tyrosine kinases involved in growth, differentiation, proliferation, survival, inflammation, metabolism and apoptosis [38, 39]. Imatinib’s antiproliferative and pro-apoptotic properties have been demonstrated in in vitro and in vivo PAH models, where imatinib induced an inversion in pulmonary vascular remodeling, reduced vascular smooth muscle cell proliferation and right ventricular hypertrophy, and improved haemodynamics [40–43].

Patients treated with oral imatinib in the phase 2 study had a significant reduction in pulmonary vascular resistance and in the phase 3 randomized controlled trial (IMPRES) significant improvements in 6-min walk distance and haemodynamics were observed in the treatment group, yet with a high rate of adverse events [44, 45]. In the phase 1 study, the dry-powder formulation of imatinib (V-101) was proved to be better tolerated in healthy adults, and targeted doses of AV-101 significantly reduced the systemic exposure of imatinib compared with oral imatinib [46].

The phase 2b/phase 3 trials are ongoing, and primary endpoints include change from baseline in Pulmonary Vascular Resistance for the phase 2b study and in Six Minute Walk Distance (6MWD) for the phase 3 study after 24 weeks of treatment [46].

Another promising ongoing phase 2b randomized double-blind controlled trial aims to evaluate the efficacy and tolerability of Treprostinil Palmitil Inhalation Powder (TPIP), an inactive prodrug of Treprostinil, expected to provide extended release of Treprostinil in the lung, with the goals of less frequent dosing (once daily) and less recurrent and severe side effects associated with the currently approved forms of Treprostinil. Endpoints include change from baseline in PVR and 6MWD at week 16 of treatment ^53^ (Table 1).

**Table 1:** Randomized clinical trials, ongoing and completed, for Group 1 PAH

Deug	Administration	Mechanism of action	Primary endpoint	Trial Phase	Ongoing /completed
Sotatercept	Subcutaneous/ Every 3 weeks	Traps activins and growth differentiation factors	1) change from baseline to week 24 in pulmonary vascular resistance 2) change from baseline at week 24 in the 6-minute walk distance	1) Phase 2 (PULSAR) 2)Phase 3 (STELLAR	Completed
Seralutinib	Inhalation/ BID	inhibition of PDGFRα/β, CSF1R, and c-KIT	Change from baseline to Week 24 in pulmonary vascular resistance by right heart catheterization	Phase 2	Completed
Imatinib	Inhalation/ BID	Inhibition of tyrosine kinase involved in growth, differentiation, proliferation, survival, inflammation, metabolism and apoptosis	1) Phase 2b: placebo corrected change in pulmonary vascular resistance (PVR) 2) Phase 3: placebo corrected change in 6- minute walk distance (6MWD)	1)Phase 2b 2) Phase 3	Ongoing
Treprostinil Palmitil	Inhalation/ OD	Increase of the prostacyclin effect on receptors	Change from baseline to Week 16 in PVR	Phase 2b	Ongoing

## Conclusions

Pulmonary arterial hypertension is still characterized by high mortality and morbidity. Data derived from studies and real-life experience suggest that a greater reduction in PVR is necessary to achieve reverse remodeling of the right ventricle in PAH, and consequently to improve clinical status and prognosis.

Therefore, early and aggressive combination therapies are needed, especially in higher risk categories.

Approved PAH therapies slow disease progression and improve life expectancy, although to date some patients do not achieve low-risk status with optimized medical therapy, and their long-term prognosis remains poor. New therapeutic targets and novel drugs are being investigated: remarkable results have derived from the phase II and III trials on Sotatercept, a subcutaneous drug with dosing every 3 weeks, and promising results are expected to come in the following years from several ongoing trials targeting new pathways for the treatment of pulmonary arterial hypertension.
